# PGR5/PGRL1 and NDH Mediate Far-Red Light-Induced Photoprotection in Response to Chilling Stress in Tomato

**DOI:** 10.3389/fpls.2020.00669

**Published:** 2020-05-27

**Authors:** Feng Wang, Jiarong Yan, Golam Jalal Ahammed, Xiujie Wang, Xin Bu, Hengzuo Xiang, Yanbing Li, Jiazhi Lu, Yufeng Liu, Hongyan Qi, Mingfang Qi, Tianlai Li

**Affiliations:** ^1^College of Horticulture, Shenyang Agricultural University, Shenyang, China; ^2^Key Laboratory of Protected Horticulture, Ministry of Education, Shenyang, China; ^3^National and Local Joint Engineering Research Center of Northern Horticultural Facilities Design and Application Technology (Liaoning), Shenyang, China; ^4^College of Forestry, Henan University of Science and Technology, Luoyang, China

**Keywords:** far-red light, PGR5/PGRL1, NDH, cyclic electron flow (CEF), cold stress, photoprotection, photoinhibition, *Solanum lycopersicum*

## Abstract

Plants experience low ambient temperature and low red to far-red ratios (L-R/FR) of light due to vegetative shading and longer twilight durations in cool seasons. Low temperature induce photoinhibition through inactivation of the photosynthetic apparatus, however, the role of light quality on photoprotection during cold stress remains poorly understood. Here, we report that L-R/FR significantly prevents the overreduction of the entire intersystem electron transfer chain and the limitation of photosystem I (PSI) acceptor side, eventually alleviating the cold-induced photoinhibition. During cold stress, L-R/FR activated cyclic electron flow (CEF), enhanced protonation of PSII subunit S (PsbS) and de-epoxidation state of the xanthophyll cycle, and promoted energy-dependent quenching (qE) component of non-photochemical quenching (NPQ), enzyme activity of Foyer-Halliwell-Asada cycle and D1 proteins accumulation. However, L-R/FR –induced photoprotection pathways were compromised in tomato *PROTON GRADIENT REGULATION5* (*PGR5*) and *PGR5-LIKE PHOTOSYNTHETIC PHENOTYPE1A* (*PGRL1A*) co-silenced plants and *NADH DEHYDROGENASE-LIKE COMPLEX M* (*NDHM*) -silenced plants during cold stress. Our results demonstrate that both PGR5/PGRL1- and NDH-dependent CEF mediate L-R/FR –induced cold tolerance by enhancing the thermal dissipation and the repair of photodamaged PSII, thereby mitigating the overreduction of electron carriers and the accumulation of reactive oxygen species. The study indicates that there is an anterograde link between photoreception and photoprotection in tomato plants during cold stress.

## Highlights

Far-red light positively regulates photoprotection through PGR5/PGRL1- and NDH- mediated cyclic electron flow in response to chilling stress in tomato.

## Introduction

Photosynthesis is the most important biosynthetic process on earth, which converts light energy into chemical energy that drives the food chain. Two types of electron transport drive the energy supply. In linear electron transport (LET), electrons derived from water splitting in photosystem II (PSII) are transported to PSI via plastoquinone (PQ), the cytochrome *b_6_f* (Cyt *b_6_f*) complex and plastocyanin (PC), which ultimately reduce NADP^+^ to NADPH. Coupled with the electron transport, the proton motive force (*pmf*) composed of a transthylakoid proton gradient (ΔpH) and a membrane potential (Δψ) is formed, resulting in the generation of ATP synthesis (Junesch and Gräber, 1991; Kramer et al., 2003; Armbruster et al., 2017). Notably, thylakoid luminal acidification slows the plastoquinol oxidation at the Cyt *b_6_f* complex to prevent excess electron flow toward PSI. This phenomenon is called photosynthetic control (Tikhonov et al., 1981). Photosynthetic control depends on the balance between proton release in the lumen and the “consumption” of these protons by ATP-synthase, and it is an important process for the regulation of photosynthetic LET. When the ATP/NADPH ratio (1.29) in LET does not satisfy the requirement of that (1.5) for CO_2_ assimilation (Allen, 2002; Shikanai, 2007; Hahn et al., 2018), cyclic electron flow (CEF) around PSI, which delivers electrons from ferredoxin (Fd) to PQ to form ΔpH without accumulation of NADPH, is driven to balance the production of ATP with NADPH for CO_2_ fixation (Yamori and Shikanai, 2016).

Temperature affects photosynthetic rate and photosynthetic electron flow (Yamori et al., 2010). When chilling-sensitive plants are transferred from optimal temperature to low temperature, a decrease in light use efficiency at low temperature can cause an increase in the excess excitation energy, which induces reactive oxygen species (ROS) production, leading to PSII and PSI photoinhibition (Zhang and Scheller, 2004; Chen et al., 2013; Huang et al., 2016; Zhang et al., 2020). PSII photoinhibition occurs when the rate of photodamage exceeds the rate of its subsequent repair (Allahverdiyeva and Aro, 2012). Unlike PSII, PSI is not frequently damaged. Once PSI is damaged, the recovery of photoinhibited PSI becomes very slow (Sonoike, 2011). Slow recovery of PSI centers has been reported in chilling-sensitive plants such as tomato (Wang et al., 2018), cucumber (Sonoike et al., 1995), tobacco (Huang et al., 2016) and sweet pepper (Li et al., 2004a). Recent studies show that electron flow from PSII to PSI could be controlled through the induction of CEF under environmental stress (Suorsa et al., 2012, 2016; Tikkanen et al., 2015; Yamori and Shikanai, 2016; Yamori et al., 2016; Huang et al., 2017), indicating that CEF may play an important role in alleviating PSI photoinhibition at low temperature stress.

For CEF-PSI, two distinct and partially redundant pathways have been suggested to exist in plant chloroplasts (Munekage et al., 2004; Shikanai, 2007). One pathway, i.e., antimycin A-insensitive pathway, is mediated by chloroplast NADH dehydrogenase-like (NDH) complex (Peltier et al., 2016). The other pathway is mediated by PROTON GRADIENT REGULATION5 (PGR5) and PGR5-like Photosynthetic Phenotype1 (PGRL1) protein complex, which is sensitive to antimycin A (Munekage et al., 2002; DalCorso et al., 2008). Both CEF pathways could move H^+^ into the thylakoid lumen via the Q-cycle in the Cyt *b_6_f* complex, while NDH-dependent CEF could extra pump H^+^ from the stroma to the thylakoid lumen (Strand et al., 2017). Lumen acidification by CEF activates the thermal dissipation of excess energy (qE), a dominant component of non-photochemical quenching (NPQ) chlorophyll fluorescence, to protect PSII against photoinhibition (Müller et al., 2001; Takahashi and Badger, 2011). Recent studies have demonstrated that acceleration of photoinhibition caused by impairment of qE is not (or less) attributed to acceleration of photodamage to PSII but more directly related to inhibition of the repair of photodamaged PSII at the step of the D1 protein synthesis (Li et al., 2002; Sarvikas et al., 2006; Takahashi et al., 2009). qE is associated with the conversion of violaxanthin (V) to zeaxanthin (Z), which depends on the catalyst violaxanthin de-epoxidase (VDE) and protonation of the PSII protein subunit PsbS in plants (Niyogi et al., 1998; Li et al., 2002; Kromdijk et al., 2016). Both these component reactions are enhanced by low lumenal pH, which is accompanied by the generation of ΔpH through LEF and CEF (Niyogi, 1999). Thus, we speculate that CEF-PSI may play dual roles in preventing plants from photoinhibition by mitigating overreduction of electron carriers, and enhancing thermal dissipation of excess energy and repair of photodamaged PSII. However, comprehensive and detailed information of these coordinated processes remains uncertain, particularly in plants exposed to stress conditions.

In nature, plants continuously experience frequent fluctuations of light caused by sunflecks, shading and seasonal changes (Pearcy, 1990). However, little information is known about how varying light quality affects photosynthetic and photoprotection processes. To perceive the light quality signals, plants have developed a set of sophisticated photoreceptors, including phytochromes (PHY), which respond to red (R) and far-red (FR) light; blue light-activated phototropins (PHOT), cryptochromes (CRY), Zeitlupe family proteins; and the ultraviolet B (UVB) photoreceptor UV Resistance locus 8 (UVR8; Möglich et al., 2010). It has been reported that light quality signals regulate chloroplast avoidance movement to reduce photodamage in plants through PHY and PHOT, and/or neochromes (Jarillo et al., 2001; Kagawa et al., 2001; Kasahara et al., 2002; Suetsugu et al., 2005, 2017; Jaedicke et al., 2012). When UV or short-wavelength green and blue lights increase, plants produce phenolics or flavonoids to scavenge ROS and reduce photodamage (Cruces et al., 2017; Liu et al., 2018). Interestingly, recent advances revealed that both UVR8-PSBS-LHCSR and PHOT-LHCSR3 pathways contribute to promoting qE and result in enhanced high light tolerance of *Chlamydomonas* (Allorent et al., 2016; Petroutsos et al., 2016; Allorent and Petroutsos, 2017; Demarsy et al., 2018), which confirmed the role played by photoperception in mediating photoprotection. We also found that phytochrome is involved in photoprotection through PGR5-dependent CEF pathway during cold stress (Wang et al., 2018). However, some results have shown that NDH-dependent CEF is also involved in plant response to various environmental stresses, such as drought (Munné-Bosch et al., 2005), high temperature (Wang et al., 2006), and low temperature (Li et al., 2004a; Yamori et al., 2011). In addition, the clear phenotypes (e.g., growth and non-photochemical chlorophyll fluorescence quenching) of NDH-deficient mutants is observed when the PGR5-PGRL1 protein-dependent pathway is also impaired in double mutants (Munekage et al., 2004; Gotoh et al., 2010), indicating that chloroplast NDH may act as a safety valve when the stroma is highly reduced. Therefore, whether NDH-dependent CEF also mediates FR regulation of photoprotection and its physiological function in tomato plants during cold stress should be clarified.

In this study, we show that low red to far-red ratios (L-R/FR) activate both PGR5/PGRL1A- and NDH-dependent CEF in tomato plants during cold stress. Reverse genetic approaches revealed that L-R/FR -induced cold tolerance is compromised in tomato *PGR5/PGRL1A-* and *NDHM-* silenced plants. PGR5/PGRL1A- and NDH-dependent CEF enhanced PsbS protein accumulation and protonation via lumen acidification, and promoted the conversion of xanthophyll cycle and thermal dissipation of excess energy. Moreover, PGR5/PGRL1A- and NDH-dependent CEF enhanced the enzymes activity in Foyer-Halliwell-Asada cycle to scavenge ROS, and alleviated the overreduction of electron carriers in photosystem to reduce photodamage. The decrease of D1 protein synthesis in *PGR5/PGRL1A-* and *NDHM-* silenced plants inhibited the repair of photodamaged PSII. Our results reveal that plants monitor the R/FR ratio and temperature signals to better adapt to cold stress through optimization of photosynthesis electron transport, which may provide a comprehensive understanding of plant growth and survival in a changing environment.

## Materials and Methods

### Plant Material and Growth Conditions

Seeds of wild type tomato (*Solanum lycopersicum* “Ailsa Craig”) were obtained from the Tomato Genetics Resource Center.^1^ Seedlings were grown in pots with a mixture of three parts peat to one part vermiculite, receiving Hoagland’s nutrient solution. The growth conditions were as follows: 12 h photoperiod, temperature of 25/20°C (day/night), and light intensity of 600 μmol m^–2^ s^–1^. The tobacco rattle virus (TRV)-based vectors (pTRV1/2) were used for the VIGS of tomato *PGR5*, *PGRL1A* and *NDHM* genes with the specific PCR-amplified primers listed in Supplementary Table S1. VIGS was performed as described in Wang et al. (2016). pTRV-*PGR5/PGRL1A* was an equal mixture of pTRV-*PGR5* and pTRV-*PGRL1A*. The inoculated plants grown under a 12 h photoperiod at 21°C.

### Cold and Light Treatments

Plants at the 4-leaf stage were used for all experiments, which were carried out in controlled environment growth chambers (Ningbo jiangnan instrument factory, Ningbo, China). Plants were grown at high red to far-red light ratios (H-R/FR) or L-R/FR conditions with an aerial temperature of 25 or 4°C for the cold treatment. The cold treatment lasted 5-day for all experiments, unless otherwise stated. For the light quality treatment, R light (λ_max_ = 660 nm, Philips) was maintained at a photosynthetic photo flux density (PPFD) of 120 μmol m^–2^ s^–1^ and FR light (λ_max_ = 735 nm, Philips) was supplemented. The R/FR ratios were 2.5 and 0.5 in H-R/FR and L-R/FR conditions, respectively. We carried out all light measurements using a Lighting Passport (Asensetek, Model No. ALP-01, Taiwan).

### Cold Tolerance Assays

Cellular membrane permeability, measured as relative electrolyte leakage (REL), was determined after the exposure of plants to cold stress, as described previously (Cao et al., 2007). Lipid peroxidation in leaves was estimated by measuring the malondialdehyde (MDA) equivalent by the 2-thiobarbituric acid (TBA) method (Sekmen et al., 2012). The percentage of plants that were viable 6-day after recovery at the optimum growth conditions was recorded. The accumulation of superoxide (O_2_^–^) and hydrogen peroxide (H_2_O_2_) in the leaves was detected using nitroblue tetrazolium (NBT) and 3,3′-diaminobenzidine (DAB) staining, respectively, as previously described (Xia et al., 2009; Ahammed et al., 2020).

### Chloroplast Ultrastructure

Small segments from the middle part of the leaves were fixed in 2.5% (v/v) glutaraldehyde in 0.1 M phosphate-buffered saline (pH 7.0) by vacuum infiltration, and treated for more than 4 h. After washing in buffer, the samples were postfixed in buffered 1% (w/v) osmium tetroxide, washed, dehydrated in an ethanol series, transferred to absolute acetone, and embedded in Spurr resin. Specimen was placed in eppendorf contained resin and heated at 70°C for more than 9 h. The specimen was sectioned in LEICA EM UC7 ultratome and the sections were stained with 2% (w/v) uranyl acetate and then 3% (w/v) alkaline lead citrate for 5–10 min, respectively. The samples were observed in transmission electron microscopy (Model H7650; Hitachi; Japan) at 75 kV.

### Chlorophyll Fluorescence Measurements

The chlorophyll fluorescence and P700 redox state measurement were determined *in vivo* by using a Dual-PAM-100 (Heinz Walz, Effeltrich, Germany). After leaves were dark-adapted for 30 min, a saturating pulse was applied to obtain maximal fluorescence and maximal P700 changes. The actinic light (AL) for measurements of chlorophyll fluorescence was 330 μmol photons m^–2^ s^–1^ (635 nm). The maximum quantum yield of PSII [Fv/Fm = (Fm - Fo)/Fm], effective quantum yield of PSII [Y(II) = (Fm′ - Fs)/Fm′], quantum yield of non-regulated energy dissipation of PSII [Y(NO) = Fs/Fm], quantum yield of regulated energy dissipation of PSII [Y(NPQ) = 1- Y(II) - Y(NO)], light-adapted maximum quantum yield of PSII [Fv′/Fm′ = (Fm′ - Fo′)/Fm′], and photochemical quenching coefficient [qP = (Fm′ - Fs)/(Fm′ - Fo′)] were calculated from the measurement of chlorophyll fluorescence, as described by Baker (2008), where Fo and Fo′ represents the minimum fluorescence in the dark-adapted state and light-adapted state, respectively. Fs is the light-adapted steady-state fluorescence, Fm and Fm′ represent the maximum fluorescence upon illumination with a pulse (600 ms) of saturating light (10,000 μmol m^–2^ s^–1^) in the dark-adapted state and light-adapted state, respectively. The parameters related to PSI are calculated as follows: Y(ND) = 1 – P700red, Y(NA) = (Pm – Pm′)/Pm, Y(I) = 1 – Y(ND) – Y(NA), where Y(ND) and Y(NA) are the quantum yields of non-photochemical energy dissipation in PSI due to donor and acceptor side limitations, respectively (Klughammer and Schreiber, 1994). Pm, which represents the maximal change in P700 signal upon quantitative transformation of P700 from the fully reduced to the fully oxidized state, was determined by applying a saturation pulse after pre-illumination with far-red light. Pm’ was determined similarly to Pm but without far-red pre-illumination. P700red, which represents the fraction of overall P700 that is reduced in a given state, was determined with the help of a saturation pulse. The electron transport rate (ETRI or ETRII) was calculated as 0.5 × abs I × Y (I) or 0.5 × abs I × Y (II), where 0.5 is the proportion of absorbed light reaching PSI or PSII, and abs I is absorbed irradiance taken as 0.84 of incident irradiance (Yamori et al., 2011). qE was simultaneously measured with the Dual-PAM-100 system (Heinz Walz) as previously described (Wang et al., 2018). The figure of maximum quantum yield of PSII (Fv/Fm) and curves of NPQ were determined with the Imaging-PAM (IMAG-MAXI; Heinz Walz) as previously described (Wang et al., 2018).

The polyphasic chlorophyll *a* fluorescence transients (OJIP) were measured using automated routines provided by the Dual-PAM software (Heinz Walz, Effeltrich, Germany). The measurements were performed on non-detached leaves that were previously adapted to the dark for 30 min and with a far-red pulse for the complete oxidation of the photosynthetic electron transport system, and the fluorescence intensity was measured for 1 s after the application of a saturating light pulse of 3,000 μmol m^–2^ s^–1^. Selected JIP-test parameters were calculated from the original data according to (Kalaji et al., 2014). The analyzed parameters were described in Supplementary Table S2.

### Measurement of Q_*A*_-Reoxidation, Q_*B*_-Reducing and Q_*B*_-Non-reducing Centres

The measurement of Q_*A*_^–^ reoxidation kinetics was performed by a single turnover flash in 100 μs to 70 s time range. Flash-induced increase and the subsequent decay of chlorophyll fluorescence yield were measured by a double-modulation fluorometer (PSI). Both actinic (20 μs) and measuring (2.5 μs) flashes were provided by red LEDs. Analysis of the fluorescence relaxation kinetics was based on the widely used model of the two-electron gate (Crofts and Wraight, 1983). The relaxation of the flash-induced increase in Chl *a* fluorescence yield reflects the reoxidation of Q_*A*_^–^ via forward electron transport to Q_*B*_ and back reactions with the S_2_ state of the oxygen evolving complexes (Cao and Govindjee, 1990). Multicomponent deconvolution of the measured curves was performed by using a fitting function with two exponential components: *Fv, corr* = A_1_ × exp (–t/T_1_) + A_2_ × exp (-t/T_2_) + A_0_, where *Fv, corr* is the variable fluorescence yield corrected for non-linearity, A_0_ to A_2_ are amplitudes, and T_1_ to T_2_ are time constants from which the half-lifetimes can be calculated as ln 2T for the exponential components (Wodala et al., 2008).

The Q_*B*_-reducing and Q_*B*_-non-reducing centers were calculated using a double hit (pulse) measurement protocol (Tomar et al., 2015). In this method, two fluorescence transients were induced by two subsequent pulses (each of 1 s duration). The first pulse was applied after a dark period long enough (30 min) to ensure reduction of all reaction centers, followed by a second pulse. The dark interval between the two pulses was short enough (500 ms) to allow only the re-opening of the Q_*B*_-reducing centers (fast opening centers), while closed centers that do not open within about 500 ms were considered as Q_*B*_-non-reducing centers (slow opening centers). Q_*B*_-non-reducing centers were calculated by the following equation: Bo = {[(Fv/Fm)−(Fv^*^/Fm^*^)]/(Fv/Fm)}×100%; Fv = Fm−Fo; Fv^*^ = Fm^*^−Fo^*^. Bo: relative amount of Q_*B*_-non-reducing PSII centers; Fv: variable fluorescence of first pulse; Fv*: variable fluorescence of second pulse; Fm: maximum fluorescence of first pulse; Fm*: maximum fluorescence of second pulse; Fo: minimum fluorescence of first pulse; Fo*: minimum fluorescence of second pulse.

### Oxidation-Reduction Kinetics of P700 and Post-illumination Chlorophyll Fluorescence

The redox kinetics of P700 (P700^+^) were measured as an absorption change at 820 nm (ΔA_820__–__860_) by using a Dual-PAM-100 instrument (Heinz Walz, Effeltrich, Germany) with an ED-101US/MD emitter-detector unit as described by Savitch et al. (2011). The transient reduction of P700^+^ signal after application of single turnover flashes (ST, 50 μs, PQ pools being oxidized) and multiple turnover flashes (MT, 300 ms, PQ pools being reduced) of white saturating light was used for estimation of the intersystem electron (e^–^) pool size (Asada et al., 1993). The complementary area between the oxidation curve of P700 after ST and MT excitation and the stationary level of P700^+^ under FR illumination represent the ST and MT areas, respectively, and were used for estimation of the functional pool size of intersystem electrons on a P700 reaction center basis that was calculated as: e^–^/P700 = MT area/ST area (Asada et al., 1992). Kinetic measurements of dark re-reduction of P700^+^ (t_1__/__2_) after turning off the far-red light is thought to reflect the extent of CEF around PSI (Savitch et al., 2011).

Post-illumination chlorophyll fluorescence (CEF around PSI) was monitored by the transient increase of dark-level chlorophyll fluorescence after actinic light (AL) illumination (330 μmol photons m^–2^ s^–1^, 635 nm) had been turned off by using a Dual-PAM-100 instrument (Walz, Effeltrich, Germany) with an ED-101US/MD emitter-detector unit (Wang et al., 2018).

### NADP^+^ and NADPH Assays

Frozen leaf material was homogenized in a mortar with either 1 mL 0.1 mol/L NaOH (for reduced forms, NADPH) or 0.1 mol/L HCI (for oxidized forms, NADP^+^), then heated for 5 min in a boiling water bath, and centrifuged at 15,000 *g* for 10 min at 4°C. Supernatants were neutralized with respectively 0.1 mol/L HCl or NaOH and centrifuged for 10 min at 4°C and 15,000 *g*. The oxidized and reduced forms of the nucleotide were determined by enzymatic cycling according to the method previously described (Matsumara and Miyachi, 1980). The enzyme reaction was stopped by addition of highly concentrated NaCl (6 M). In these conditions, formazan that precipitates is sedimented by centrifugation (15 min at 15,000 *g*) and solubilized in ethanol. The procedure was performed according to the previous method (Gibon and Larher, 1997).

### Activity of Antioxidant Enzymes and Xanthophyll Cycle Pigment Analysis

The activity of SOD, APX, MDAR, DHAR, and GR was measured following the protocol used as previously described (Wang et al., 2018). Xanthophyll cycle pigments were analyzed using a C30 column (YMC) equipped for HPLC (Waters) as described previously (Wang et al., 2018). The de-epoxidation state of the xanthophyll cycle pigments is defined as the (A + Z)/(V + A + Z) ratio, where A, Z, and V are the concentrations of antheraxanthin, zeaxanthin, and violaxanthin, respectively.

### RNA Extraction and qRT-PCR Analysis

Total RNA was extracted from tomato leaves using an RNAprep Pure Plant Kit (Tiangen Biotech) following the manufacturer’s recommendations. The extracted RNA was reverse-transcribed using a ReverTra Ace qPCR RT Kit with an enzyme for genomic DNA removal (Toyobo). qRT-PCR experiments were performed on an Applied Biosystems 7500 Real-Time PCR System with a SYBR Green PCR Master Mix Kit (TaKaRa). The PCR was run at 95°C for 3 min, followed by 40 cycles of 30 s at 95°C, 30 s at 58°C, and 1 min at 72°C. The tomato ACTIN gene was used as an internal control. Primers sequence was in Supplementary Table S1. The relative gene expression was calculated as described previously (Livak and Schmittgen, 2001).

### Immunoblot Analysis

Protein extraction and immunoblot analysis were performed as previously described (Wang et al., 2016, 2018, 2019). After quantification of total protein concentrations, samples of 50 mg protein were separated by SDS-PAGE electrophoresis. Proteins were then transferred onto nitrocellulose membranes (BioRad), which were then incubated with antibodies against PsbS (AS09533; Agrisera, Sweden) or D1 (AS05084; Agrisera, Sweden). After incubation with secondary anti-rabbit antibodies (Cell Signaling Technology 7074; Danvers, MA, United States), enhanced chemical luminescence (ECL) was performed to detect labeled proteins.

### Statistical Analysis

All data were statistically analyzed with SPSS software. When interaction terms were significant (*P* < 0.05), differences between means were analyzed using Tukey’s comparisons. Significant differences between treatment means are indicated by different letters.

## Results

### L-R/FR Enhances Cold Tolerance in Tomato via Alleviating ROS Accumulation and Chloroplast Structure Distortions

**FIGURE 1:**
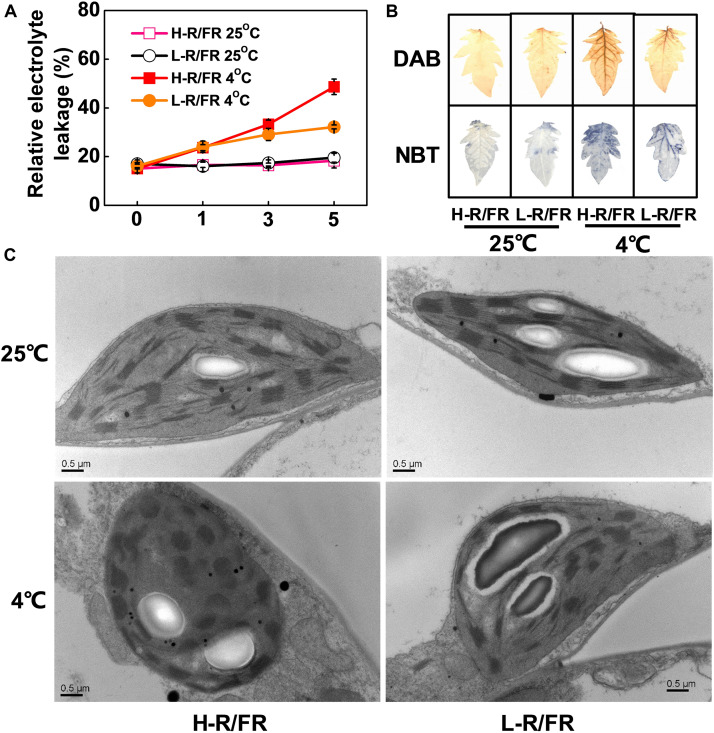
The relative electrolyte leakage, ROS accumulation and chloroplast structure in tomato plants under cold stress at different R/FR ratio conditions. **(A)** Changes of relative electrolyte leakage in tomato leaves that were exposed to high red to far-red light ratios (H-R/FR, 2.5) or low red to far-red light ratios (L-R/FR, 0.5) at 25 or 4°C for 5-day. **(B)** The accumulation of hydrogen peroxide [3,3′-diaminobenzidine (DAB) staining] and superoxide [nitroblue tetrazolium (NBT) staining] in tomato leaves that were exposed to H-R/FR or L-R/FR conditions at 25 or 4°C for 5-day. **(C)** Chloroplast structure in tomato plants grown under H-R/FR or L-R/FR conditions at 25 or 4°C for 5-day. For light-quality treatments, plants were maintained at R conditions (120 μmol m^–2^ s^–1^) and supplemented with different intensities of FR. Scale bars = 0.5 μm. Data are presented as the means of three biological replicates (±SD). Different letters indicate significant differences (*P* < 0.05) according to Tukey’s test.

To investigate the possible involvement of different R/FR ratios in the response of plants to cold stress, we measured the relative electrolyte leakage (REL) in tomato plants under cold stress. We found that REL was increased in tomato plants by cold stress, but this increase was attenuated when plants exposed to L-R/FR conditions (Figure 1A). As excessive production of reactive oxygen species (ROS) in chloroplasts causes serious damage to the photosynthetic machinery of plants, we examined the ROS (superoxide and hydrogen peroxide) and malondialdehyde (MDA) accumulation in the leaves under H-R/FR and L-R/FR conditions at 25 or 4°C (Figure 1B and Supplementary Figure S1). We found that exposure to cold stress resulted in an increased of ROS and MDA, however, L-R/FR alleviated the accumulation of ROS and MDA in tomato under cold stress.

We then analyzed the changes of plastid morphology in tomato plants under H-R/FR and L-R/FR conditions by transmission electron microscopy. There were a well-developed thylakoid membrane system composed of well-organized granal stacks and connected by stroma lamellae in tomato chloroplasts at optimal temperature conditions (Figure 1C). Cold stress promoted the distortions of plastids at both light conditions, especially the plants under H-R/FR conditions (Figure 1C). In addition, the chloroplasts contained large amounts of osmiophilic lipid droplets, which were big and round as well as presented in great numbers, in plants under H-R/FR conditions during cold stress. These results suggest that L-R/FR alleviates the damage of the membrane and chloroplast ultrastructure in tomato plants via decreasing the levels of ROS and MDA during cold stress.

### L-R/FR Protects PSII and PSI From Cold-Induced Photoinhibition

**FIGURE 2:**
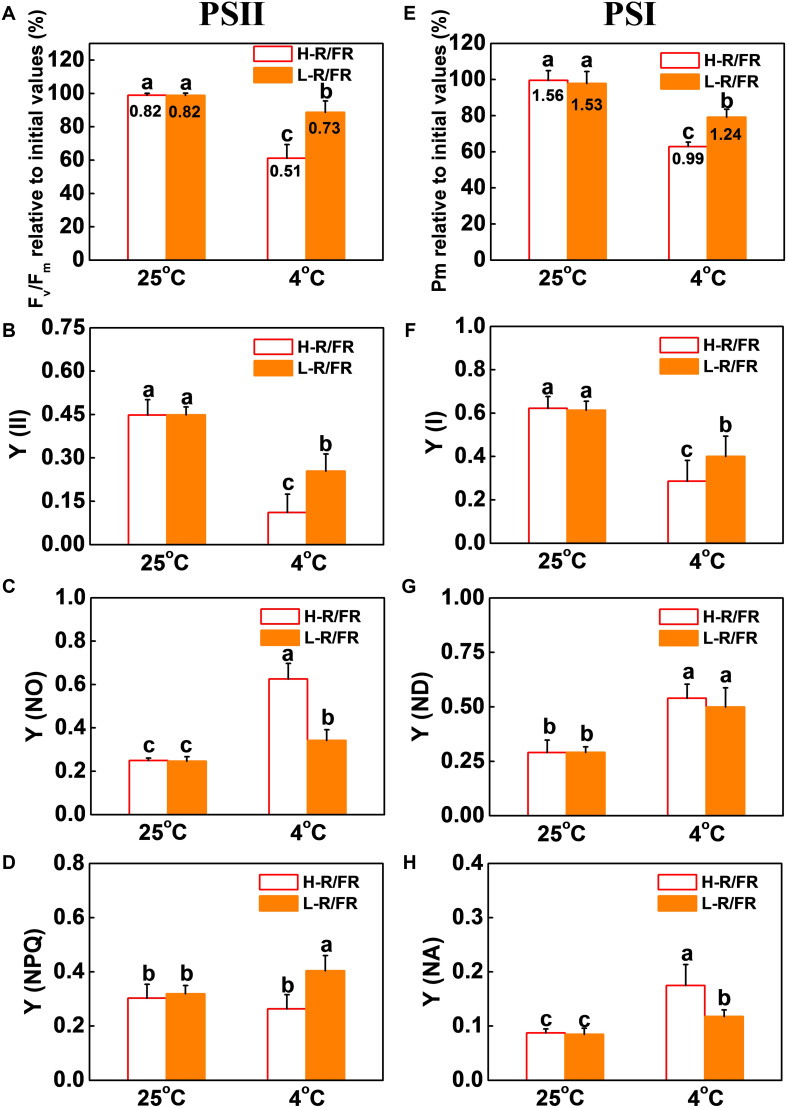
L-R/FR alleviated the inhibitory effects of cold stress on photosystem II and photosystem I in tomato plants. **(A–D)** Changes in PSII related parameters, including Fv/Fm **(A)**, Y(II) **(B)**, Y(NO) **(C)**, Y(NPQ) **(D)** in tomato plants grown at high red to far-red light ratios (H-R/FR, 2.5) or low red to far-red light ratios (L-R/FR, 0.5) conditions after exposed to 25 or 4°C for 5-day. **(E–H)** Changes in PSI related parameters, including Pm **(E)**, Y(I) **(F)**, Y(ND) **(G)**, Y(NA) **(H)** in tomato plants grown at H-R/FR or L-R/FR conditions after exposed to 25 or 4°C for 5-day. While graphs were prepared based on the values of Fv/Fm and Pm relative to the initial values before the cold and light treatments and presented in percentages, the absolute values were simultaneously shown at the top of respective bars. For light-quality treatments, plants were maintained at R conditions (120 μmol m^–2^ s^–1^) and supplemented with different intensities of FR. Data are presented as the means of three biological replicates (±SD). Different letters indicate significant differences (*P* < 0.05) according to Tukey’s test.

To examine the cold-induced photoinhibition, the photoinhibition in PSII and PSI was evaluated by measuring the Fv/Fm and Pm after the cold stress (Figures 2A,E). Cold stress decreased the Fv/Fm and Pm in plants by 61 and 63%, respectively compared with that at 25°C under H-R/FR, respectively. However, L-R/FR significantly alleviated the decrease of Fv/Fm and Pm during cold stress. While the Y(II) and ETRII at 4°C significantly decreased under both light conditions as compared with that at 25°C, the plants under L-R/FR conditions exhibited higher Y(II) and ETRII values than plants under H-R/FR conditions (Figure 2B and Supplementary Figure S2C). Since the Y(II) is determined by the Fv′/Fm′ and the qP simultaneously, we then examined the Fv′/Fm′ and qP. Both Fv′/Fm′ and qP were similar at 25°C under H-R/FR and L-R/FR conditions, but they were significantly decreased under cold stress, especially under H-R/FR conditions (Supplementary Figures S2A,B). We further found that qP decreased more rapidly compared to Fv′/Fm′, which indicated that the decrease in the Y(II) at low temperature was mainly due to the decrease in qP. Obviously, L-R/FR -induced the Y(NPQ) under cold stress (Figure 2D). In contrast, the Y(NO) at 4°C was higher than that at 25°C, especially under H-R/FR conditions (Figure 2C). We also found the Y(I) and ETRI were significantly lower under cold stress than that at 25°C, especially under H-R/FR conditions (Figure 2F and Supplementary Figure S2D). Interestingly, low temperature induced the quantum yield of PSI non-photochemical quenching due to the donor side limitation [Y(ND)], but there was no difference at different R/FR ratio conditions (Figure 2G). However, L-R/FR alleviated the increase of PSI acceptor side limitation [Y(NA)] under cold stress (Figure 2H). These results suggest that both photochemical energy conversion and protective regulatory mechanisms in PSII and PSI are inefficient under cold stress, but L-R/FR could alleviate photoinhibition by enhancing energy dissipation in PSII and decreasing PSI acceptor-side limitations and overreduction of electron carriers in tomato plants.

### Effects of L-R/FR on the Electron Transport and Energy Distribution in Photosynthetic Response Under Cold Stress

**FIGURE 3:**
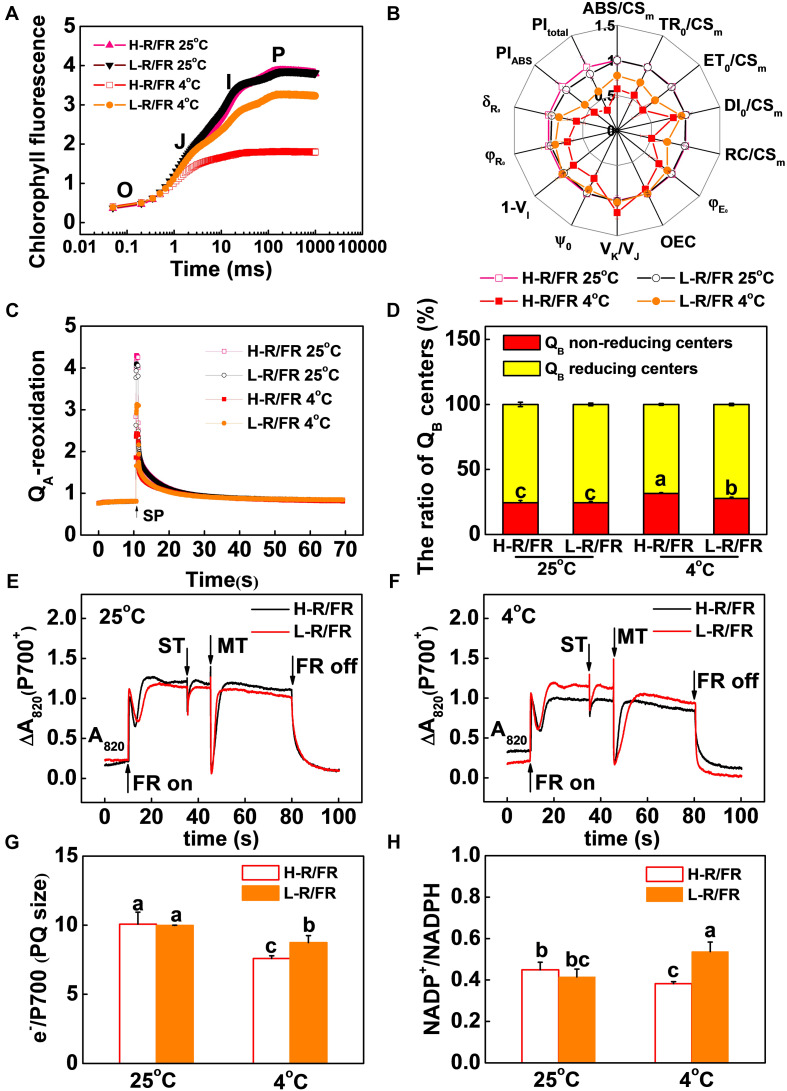
Effects of light-quality on the electron absorbed, transport and energy distribution in photosynthetic response under cold stress. **(A)** OJIP transients recorded from tomato leaves grown at high red to far-red light ratios (H-R/FR, 2.5) or low red to far-red light ratios (L-R/FR, 0.5) conditions after exposed to 25 or 4°C for 5-day. **(B)** Radar plot presentation of some physiological parameters derived from JIP-test quantifying PSII behavior of tomato leaves exposed to 25 or 4°C for 5-day under H-R/FR or L-R/FR conditions. **(C,D)** Q_A_-reoxidation **(C)**, Q_B_ reducing and non-reducing side centers of PSII **(D)** in tomato plants after exposed to 25 or 4°C for 5-day under H-R/FR or L-R/FR conditions. **(E–H)** Typical trace of light-induced P700 transients measured as Δ820 **(E,F)**, intersystem electron pool size (e-/P700, MT_AREA_/ST_AREA_; **G**), and NADP^+^/NADPH ratios **(H)** in tomato plants after exposed to 25 or 4°C for 5-day under H-R/FR or L-R/FR conditions. For light-quality treatments, plants were maintained at R conditions (120 μmol m^–2^ s^–1^) and supplemented with different intensities of FR. Data are presented as the means of three biological replicates (±SD). Different letters indicate significant differences (*P* < 0.05) according to Tukey’s test.

OJIP transients can provide information on the absorption, distribution and utilization of energy in photosynthesis. We found that the R/FR ratios had no significant effects on the shapes of the curves at 25°C (Figure 3A). In contrast, the cold treatment under H-R/FR conditions decreased the relative fluorescence intensity at J to P step, which demonstrated that plants suffered serious injury. However, L-R/FR alleviated the decreased fluorescence intensity (Figure 3A).

In parallel with the OJIP curve, light quality -induced changes in biophysical parameters under cold stress were analyzed by the OJIP-test (Figure 3B and Supplementary Table S2). The ABS/CS_m_, TR_0_/CS_m_, ET_0_/CS_m_ and DI_0_/CS_m_ exhibited significantly decrease after cold stress, especially under H-R/FR conditions. L-R/FR significantly attenuated the decrease of these parameters by increasing the density of active RCs (RC/CS_m_), which represented as Q_A_^–^ reducing PSII reaction centers (Figure 3B). The significant decrease of ψ_0_ and φ_E__0_ in the leaves of stressed plants suggested that the electron transport beyond Q_A_^–^ and from Q_A_^–^ to the intersystem of electron acceptors decreased under cold stress (Figure 3B). To further understand the effect of light quality on the function of the acceptor side of PSII of tomato, the Q_A_^–^ reoxidation kinetic curves of the plants grown under 25 and 4°C with H-R/FR or L-R/FR were measured (Figure 3C). It can be clearly seen that light quality had no effect on the amplitudes of fast (shown by A_1_) or slow components (shown by A_2_) at 25°C (Table 1). The amplitudes of fast components decreased while the amplitudes of slow components increased by the exposure of plants to cold stress. Furthermore, the amplitudes of fast components and slow components were 10.5% lower in the plants under L-R/FR than plants under H-R/FR conditions (Table 1). Meanwhile, cold stress -induced a significant increase in Q_B_-non-reducing centers, especially in plants grown under H-R/FR conditions (Figure 3D). This result showed that Q_A_^–^ to Q_B_/Q_B_^–^ electron transfer was hindered during cold stress, which resulted in much more Q_A_^–^ reoxidation through reverse reactions with the S_2_ state of the OEC, i.e., S_2_ (Q_A_Q_B_)^–^ charge recombination. However, low temperature obviously increased the V_K_/V_J_ under H-R/FR conditions, indicating an additional limitation probably linked to the OEC (Figure 3B). These results indicated that the structure and function of the PSII were damaged due to the significant decrease in PI_ABS_ after cold stress, especially under H-R/FR conditions (Figure 3B).

**TABLE 1:** Kinetic deconvolution of fluorescence decay kinetics of tomato during cold stress.

Treatment	Fast component		Slow component	
		
	A_1_ (%)	T_1_ (s)	A_2_ (%)	T_2_ (s)
H-R/FR 25°C	70.2	8.342	29.8	0.467
L-R/FR 25°C	68.7	8.244	31.3	0.470
H-R/FR 4°C	54.1	11.424	45.9	0.525
L-R/FR 4°C	63.1	9.203	36.9	0.593

The lack of I-step and a decrease in J-P in the OJIP pattern reflect the damage of plastoquinone pool (PQ) and electron transport from PQH_2_ to PSI (Frydenvang et al., 2015). To assess the extent of PSI photooxidation (P700^+^), the extent of FR light-induced changes in absorbance at 820 nm (ΔA820) was measured (Figures 3E,F). The results showed that much higher capacity for P700 photooxidation (Δ820, P700^+^) in cold-stressed leaves under L-R/FR conditions compared with that in plants under H-R/FR conditions (Figure 3F). Moreover, the estimates of e-/P700 (PQ size) indicated 15.2% larger electron donor pool to PSI in plants under L-R/FR relative to plants under H-R/FR during cold stress (Figure 3G). Additionally, we found that the parameters related to electron uptake from the PSI acceptors, i.e., φ_R__0_, δ_Ro_, 1-V_I_ and PI_total_ decreased after cold stress, especially under H-R/FR conditions (Figure 3B). Meanwhile, after exposure of plants to cold conditions, the contents of NADP^+^ and ratio of NADP^+^/NADPH decreased under H-R/FR conditions while that increased under L-R/FR conditions (Figure 3H and Supplementary Figure S3). These results suggest that L-R/FR alleviates the limitation of PSI acceptor side during cold stress in tomato plants by regulating the electron transport, and the reduction and oxidation of PQ, which depend on the efficiency of the electron uptake from the PSI acceptors and the number of available oxidized forms of NADP.

### Both PGR5/PGRL1- and NDH-Dependent CEF Are Essential for L-R/FR -Induced Photoprotection in Tomato Plants During Cold Stress

**FIGURE 4:**
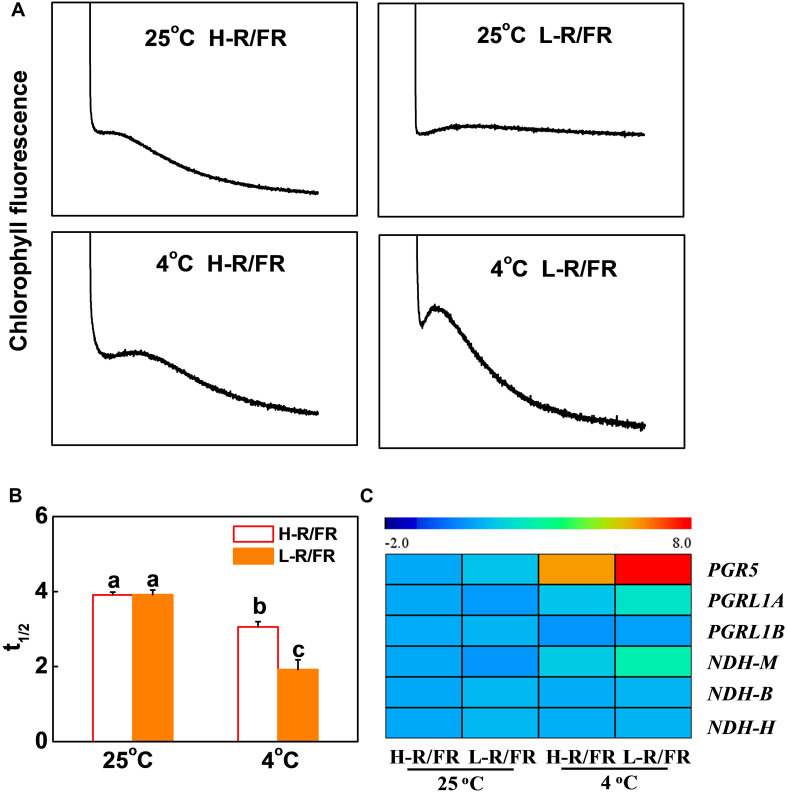
L-R/FR induced cyclic electron flow (CEF) in tomato plants under cold stress. **(A)** Post-illumination chlorophyll fluorescence transients in tomato plants grown at high red to far-red light ratios (H-R/FR, 2.5) or low red to far-red light ratios (L-R/FR, 0.5) conditions after exposed to 25 or 4°C for 5-day. **(B)** Reduction of P700^+^ (t_1__/__2_, P700red) in tomato plants grown at H-R/FR or L-R/FR conditions after exposed to 25 or 4°C for 5-day. **(C)** Heatmap showing gene expression of PGR5/PGRL1- and NdH-dependent CEF in tomato plants grown at H-R/FR or L-R/FR conditions after exposed to 25 or 4°C for 6 h. For light-quality treatments, plants were maintained at R conditions (120 μmol m^–2^ s^–1^) and supplemented with different intensities of FR. Data are presented as the means of three biological replicates (±SD). Different letters indicate significant differences (*P* < 0.05) according to Tukey’s test.

In Supplementary Figure S2E, the results showed that the values of ETRI-ETRII under L-R/FR were higher than those under H-R/FR during cold stress. To examine whether CEF could alleviate PSI acceptor-side limitation via electron transport, a transient post-illumination, which is considered to involve CEF in PSI mediated by NDH complex (Yamori et al., 2011), and the t_1__/__2_ of P700^+^ re-reduction, which reflects the CEF induction rate around PSI (Savitch et al., 2011), were investigated in tomato (Supplementary Figure S4). At 25°C, the post-illumination fluorescence rise was not detected in tomato plants (Figure 4A). Cold stress slightly enhanced the post-illumination fluorescence rise in plants under H-R/FR, but the enhancement was lower in comparison with plants under L-R/FR. Furthermore, in comparison to plants grown under optimal temperature, the t_1__/__2_ of P700^+^ re-reduction was faster in plants after cold stress, especially that in plants under L-R/FR (Figures 3F, 4B). These results suggested that L-R/FR induced CEF in tomato plants during cold stress.

We then investigated the transcript levels of CEF-related genes, including *PGR5*, *PGRL1A*, *PGRL1B*, *NDHM*, *NDHB* and *NDHH* in tomato leaves (Figure 4C). Among these genes, the increases in the transcript levels of *PGR5*, *PGRL1A* and *NDHM* were greater in L-R/FR conditions than that in H-R/FR conditions under cold stress (Figure 4C), which indicated that both PGR5/PGRL1- and NDH-dependent CEF might be involved in the light-quality regulated photoprotection during cold stress.

**FIGURE 5:**
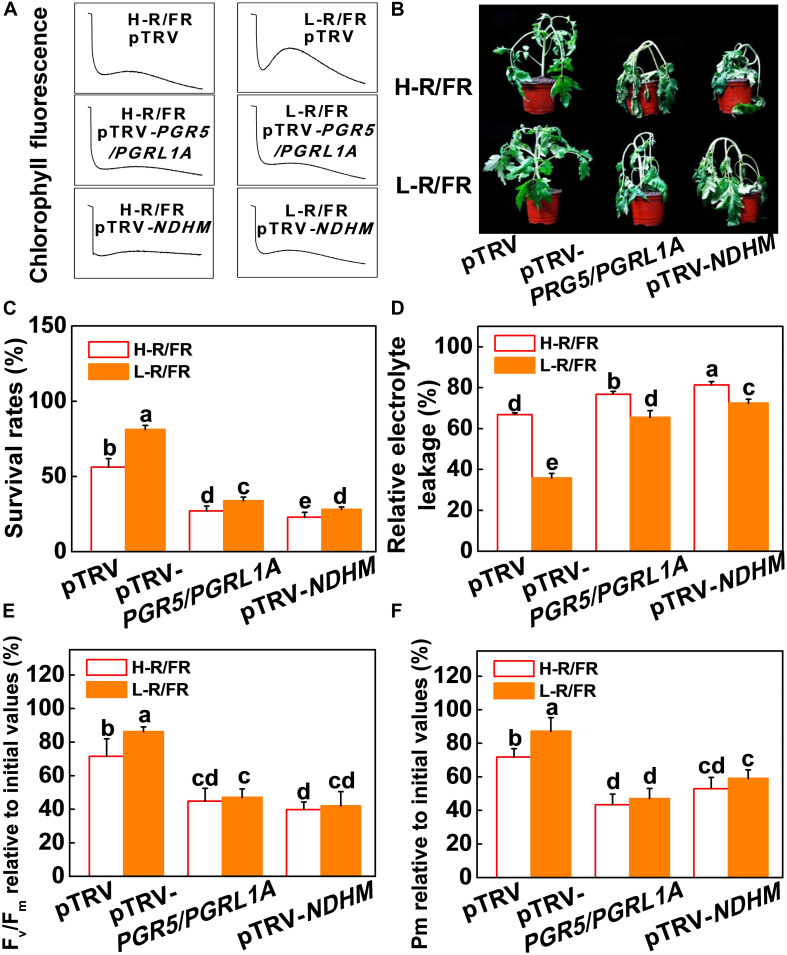
PGR5/PGRL1- and NdH-dependent CEF were essential for L-R/FR alleviating cold stress –induced photoinhibition in tomato plants. **(A)** Post-illumination chlorophyll fluorescence (CEF around PSI) in wild-type (pTRV), *PGR5* and *PGRL1A* co-silenced plants (pTRV-*PGR5/PGRL1A*), and *NDHM*-silenced plants (pTRV-*NDHM*) after exposure to 4°C for 5-day under high red to far-red light ratios (H-R/FR, 2.5) or low red to far-red light ratios (L-R/FR, 0.5) conditions. **(B–D)** Phenotypes **(B)**, survival rates **(C)** and relative electrolyte leakage **(D)** in pTRV, pTRV-*PGR5/PGRL1A* and pTRV-*NDHM* plants after exposed to 4°C for 7-day under H-R/FR or L-R/FR conditions. **(E,F)** Fv/Fm **(E)** and Pm **(F)** in pTRV, pTRV-*PGR5/PGRL1A* and pTRV-*NDHM* plants after exposed to 4°C for 7-day under H-R/FR or L-R/FR conditions. Fv/Fm and Pm relative to the initial values before the cold and light treatments are shown. For light-quality treatments, plants were maintained at R conditions (120 μmol m^–2^ s^–1^) and supplemented with different intensities of FR. Data are presented as the means of three biological replicates (±SD). Different letters indicate significant differences (*P* < 0.05) according to Tukey’s test.

We then generated *PGR5/PGRL1A* co-silenced (pTRV-*PGR5/PGRL1A*) and *NDHM* (pTRV-*NDHM*) silenced tomato plants using a virus-induced gene silencing approach (VIGS; Supplementary Figure S5A). The respective silenced lines showed 68.5% and 78.4% reduction in the transcript levels of *PGR5* and *PGRL1A*, respectively, in pTRV-*PGR5/PGRL1A* plants, and a decrease of 73.3% in the transcript levels of *NDHM* in pTRV-*NDHM* plants compared with the control plants (pTRV; Supplementary Figure S5B). We found that L-R/FR-induced post-illumination fluorescence rise in pTRV plants under cold stress was not detected in the pTRV-*PGR5/PGRL1A* and pTRV-*NDHM* plants (Figure 5A). The pTRV-*PGR5/PGRL1A* and pTRV-*NDHM* plants showed an increased sensitivity to cold stress-induced photoinhibition compared to the pTRV plants, as evidenced by a decrease of survival rates, Fv/Fm, Pm, Y(II) and Y(I), as well as an increase in wilting symptoms and REL (Figures 5B–F and Supplementary Figures S6A,B). Interestingly, the L-R/FR -induced cold tolerance and alleviation of photoinhibition observed in the pTRV plants obviously decreased in the *PGR5/PGRL1A*-silenced plants and *NDHM*-silenced plants, which showed no significant differences in Fv/Fm, Pm, Y(II) and Y(I) under cold stress at both light quality regimes (Figures 5B–F and Supplementary Figures S6A,B). The Y(ND) values were lower in the *PGR5/PGRL1A*-silenced plants and *NDHM*-silenced plants than those in the pTRV plants after cold stress in both light conditions (Supplementary Figure S6C). L-R/FR alleviated the acceptor-side limitation of PSI via negatively regulating the Y(NA) parameter (Supplementary Figure S6D). However, the Y(NA) values were higher in the *PGR5/PGRL1A*-silenced plants and *NDHM*-silenced plants than those in the pTRV plants after cold stress in both light conditions (Supplementary Figure S6D). These observations clearly indicate that loss of *PGR5/PGRL1A* and *NDHM* functions compromised the L-R/FR -alleviated photoinhibition partially through the enhanced acceptor-side limitation of PSI in tomato plants under cold stress.

**FIGURE 6:**
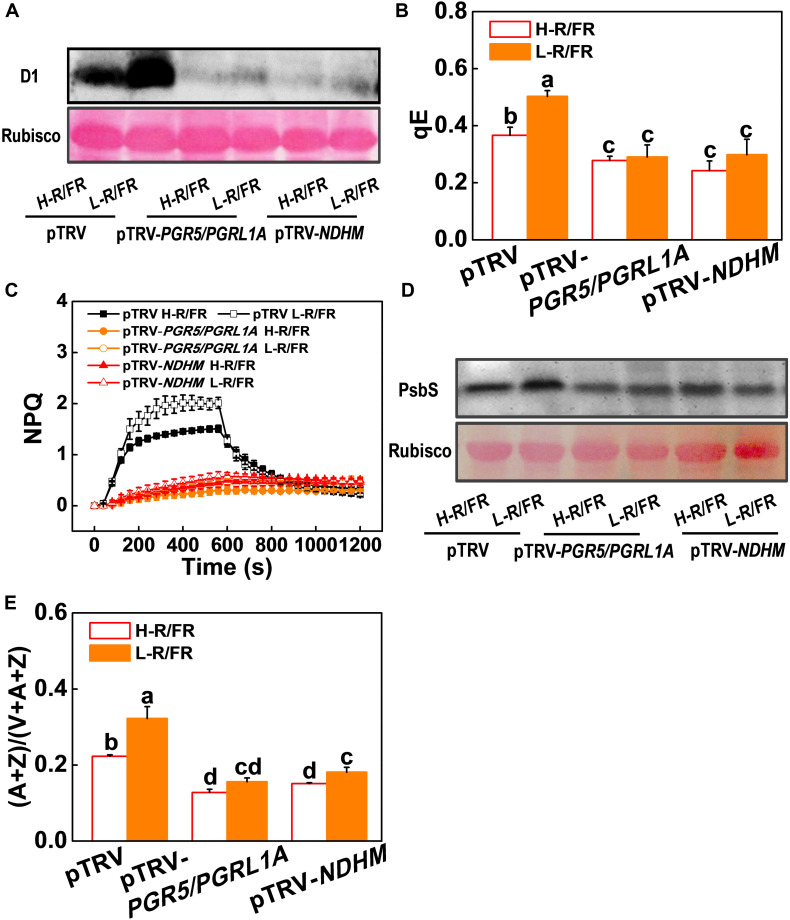
Roles of *PGR5/PGRL1A* and *NDHM* in L-R/FR induced photoprotection in tomato plants during cold stress. **(A,D)** D1 protein **(A)** and PsbS protein **(D)** in wild-type (pTRV), *PGR5* and *PGRL1A* co-silenced plants (pTRV-*PGR5/PGRL1A*), and *NDHM*-silenced plants (pTRV-*NDHM*) after exposure to 4°C for 1-day under high red to far-red light ratios (H-R/FR, 2.5) or low red to far-red light ratios (L-R/FR, 0.5) conditions. **(B,C)** qE **(B)** and NPQ **(C)** in pTRV, pTRV-*PGR5/PGRL1A* and pTRV-*NDHM* plants after exposed to 4°C for 3-day under H-R/FR or L-R/FR conditions. **(E)** The de-epoxidation state of the xanthophyll cycle, i.e., (A + Z)/(V + A + Z), in pTRV, pTRV-*PGR5/PGRL1A* and pTRV-*NDHM* plants after exposed to 4°C for 3-day under H-R/FR or L-R/FR conditions. For light-quality treatments, plants were maintained at R conditions (120 μmol m^–2^ s^–1^) and supplemented with different intensities of FR. Data are presented as the means of three biological replicates (±SD). Different letters indicate significant differences (*P* < 0.05) according to Tukey’s test.

**FIGURE 7:**
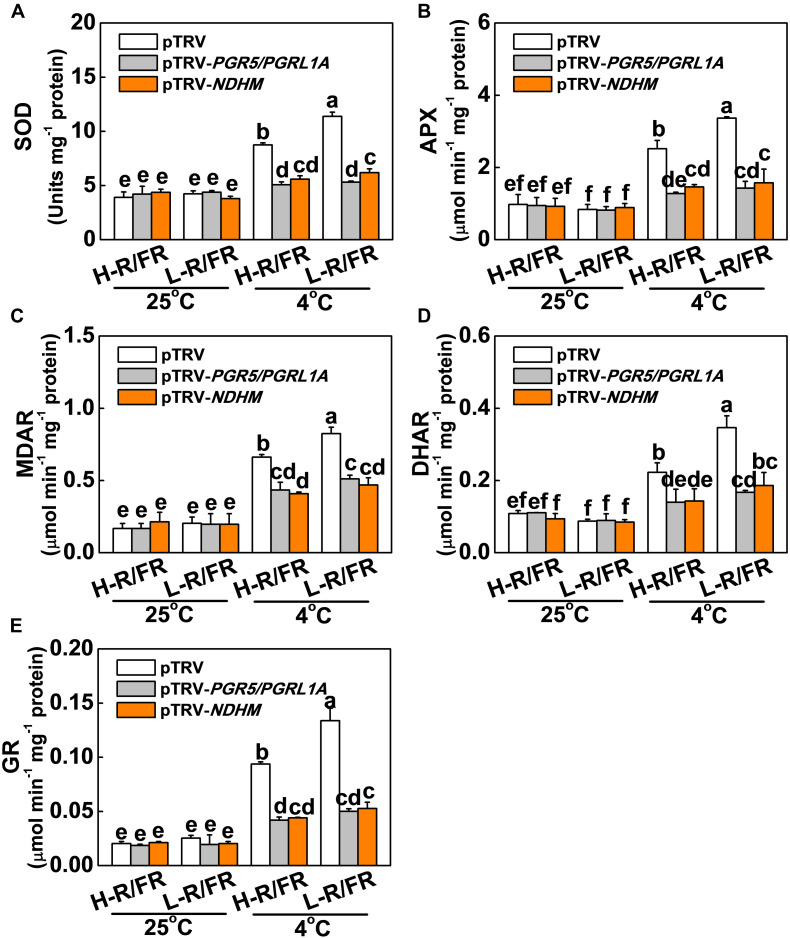
Roles of *PGR5/PGRL1A* and *NDHM* in light-quality regulated activity of antioxidant enzymes in tomato plants. **(A–E)** Activity of antioxidant enzymes (SOD, **A**; APX, **B**; MDAR, **C**; DHAR, **D**; and GR, **E**) involved in Foyer-Halliwell-Asada cycle after the wild-type (pTRV), *PGR5* and *PGRL1A* co-silenced plants (pTRV-*PGR5/PGRL1A)*, and *NDHM*-silenced plants (pTRV*-NDHM*) exposed to 25 or 4°C under H-R/FR or L-R/FR light conditions for 3-day. For light-quality treatments, plants were maintained at R conditions (120 μmol m^–2^ s^–1^) and supplemented with different intensities of FR. Data are presented as the means of three biological replicates (±SD). Different letters indicate significant differences (*P* < 0.05) according to Tukey’s test.

The roles of NPQ, CEF, Foyer-Halliwell-Asada cycle and the repair cycle for damaged PSII (primarily the D1 protein) reaction centers in photoprotection are well established (Foyer et al., 1995; Murchie and Niyogi, 2011; Takahashi and Badger, 2011; Chen and Gallie, 2012). However, whether PGR5-PGRL1- and NDH-dependent CEF induced photoprotection through these pathways is little known. Here, we found that levels of D1 protein accumulation, qE, NPQ, de-epoxidation state of the xanthophyll cycle, i.e., (A + Z)/(V + A + Z) ratio, PsbS protein accumulation and antioxidant enzymes (SOD, APX, MDAR, DHAR and GR) activity significantly decreased in pTRV-*PGR5/PGRL1A* and pTRV-*NDHM* plants compared with those in pTRV plants under cold stress (Figures 6, 7). A decrease of R/FR ratios significantly increased the accumulation of D1 and PsbS proteins, and levels of qE and NPQ, as well as the ratio of (A + Z)/(V + A + Z), and the activity of those antioxidant enzymes in tomato pTRV plants (Figures 6, 7). However, L-R/FR -induced changes of these photoprotection parameters were abolished or attenuated in the pTRV-*PGR5/PGRL1A* and pTRV-*NDHM* plants (Figures 6, 7). These results suggest that PGR5/PGRL1- and NDH-dependent CEF in tomato plants plays a dual roles in L-R/FR -induced cold tolerance. PGR5/PGRL1- and NDH-dependent CEF not only alleviates PSI acceptor-side limitations and overreduction of electron carriers, but also induces the protonation of PsbS and conversion of xanthophyll carotenoids to rapidly activate qE and NPQ for thermal dissipation. Moreover, it generates ATP to promote D1 protein synthesis for repairing the damaged PSII.

## Discussion

In natural environment, the decrease in temperature is often associated with longer twilight durations during autumn at northern latitudes, which is characterized by a significant drop in R/FR ratio. Monitoring of R/FR ratio signals would provide an early warning and confer some protection to plants subject to sudden decrease in temperature during night and seasonal variations (Wang et al., 2019, 2020). Optimization of photosynthesis electron transport is critical for alleviation of cold stress-induced photoinhibition. Thus, it is crucial to understand whether changes of R/FR ratios regulate plant cold tolerance via modulating photosynthetic electron transport processes and photoprotection. In this study, we found that photoinhibition was exacerbated by cold stress, as evidenced by a decrease of Fv/Fm, Pm, PI_ABS_ and PI_total_ (Figures 2A,E, 3B). Low temperature inhibited the absorption, distribution and utilization of energy in photosynthesis by reducing the ABS/CS_m_, TR_0_/CS_m_, ET_0_/CS_m_ and DI_0_/CS_m_ (Figure 3B), while L-R/FR significantly attenuated the decrease of these parameters by increasing the density of active RCs (RC/CS_m_), i.e., Q_A_^–^ reducing PSII reaction centers. The increase in the number of Q_A_^–^ reoxidation and Q_B_-non-reducing centers under cold stress indicated that photosynthetic electrons transport had been partially blocked from Q_A_ to Q_B_ in PSII (Figures 3C,D and Table 1). Recent studies suggest that moderate phosphorylation of PSII and LHCII allows the PSI complexes to move toward the grana margins and transfer adequate excitation energy to PSI, which alleviates the PSII photoinhibition (Tikkanen et al., 2010; Grieco et al., 2012; Tikkanen and Aro, 2014). However, controlled photoinhibition of PSII can protect PSI from photoinhibition by regulating the electron transport chain and preventing the formation of ROS (Tikkanen et al., 2014). Low temperature damaged the PQ and electron transport from PQH_2_ to PSI (Figure 3G; Frydenvang et al., 2015), which resulted in an imbalance between light reactions and electron consumption in the chloroplast, leading to an overreduction of electron carriers (including Q_A_^–^ and Q_B_^–^ reducing centers, PQ and PSI) and generation of ROS (Figure 1A; Foyer et al., 2012). Interestingly, L-R/FR promoted NADP^+^/NADPH ratio and the number of available oxidized forms of NADP to alleviate PSI acceptor side limitation (Figures 2H, 3H and Supplementary Figure S3). Recent studies also showed that FR could enhance the photochemical efficiency and suppress the fluctuating light-induced PSI photodamage (Kono et al., 2017; Krahmer et al., 2018; Zhen et al., 2018). These results suggest that L-R/FR can alleviate cold stress –induced photoinhibition by modulating the photosynthetic electron transport.

There is now clear genetic and molecular evidence for the occurrence of at least two distinct CEF pathways in angiosperms: the PGR5/PGRL1 and the NDH pathways (Munekage et al., 2004; Yamori and Shikanai, 2016). The role of PGR5/PGRL1-dependent CEF in photosynthetic regulation under various environments has been extensively studied. The *pgr5* or *pgrl1* mutants show sensitivity to high light (Munekage et al., 2002, 2004; Takahashi et al., 2009), fluctuating light (Suorsa et al., 2012; Kukuczka et al., 2014), high temperature (Zhang and Sharkey, 2009), and low CO_2_ concentrations (Munekage et al., 2008). It has long been recognized that the NDH pathway is a minor pathway for the activation of CEF, because the rate of NDH-dependent CEF is too low to appreciably contribute to proton gradient formation (Munekage et al., 2002, 2004). However, recent results have showed that NDH-dependent CEF is involved in plant response to various environmental stresses, such as extremely strong light (Endo et al., 1999; Takabayashi et al., 2002), drought (Munné-Bosch et al., 2005), low humidity (Horváth et al., 2000), high temperature (Wang et al., 2006; Zhang and Sharkey, 2009), and low temperature (Li et al., 2004a; Yamori et al., 2011). As inhibition of CO_2_ assimilation could lead to overreduction of the electron transport chain, NDH-dependent CEF has been proposed to prevent overreduction of the stroma, especially under stress conditions (Rumeau et al., 2007; Shikanai, 2007). Here, we found that low temperature activated genes expression of *PGR5*, *PGRL1A*, and *NDHM*, and induced CEF, especially under L-R/FR conditions (Figure 4). When we silenced the *PGR5/PGRL1A* and *NDHM* in tomato plants, the L-R/FR-induced post-illumination fluorescence rise was not detected during cold stress (Figure 5A). Gotoh et al. (2010) also found the post-illumination transient can be induced by the Fd- (or PGR5)-dependent PQ reduction. In response to sink limitation of linear electron flow, the main route for the PSI CEF maybe shifted from the NDH-dependent path to the Fd-dependent path. Meanwhile, both *PGR5/PGRL1A*- and *NDHM*-silenced plants showed a reduced cold tolerance, and the alleviatory effect of L-R/FR on photoinhibition was almost disappeared in these tomato silenced plants (Figures 5B–F). Therefore, we conclude that both PGR5/PGRL1- and NDH-dependent CEF-PSI in tomato plants have physiological roles in L-R/FR -alleviated photodamage during cold stress (Figure 5). In agreement with our results, recently, Yamori et al. (2016) also showed that both PGR5-dependent and NDH-dependent CEF-PSI have physiological roles in sustaining photosynthesis and growth of rice under fluctuating light. In addition, Arabidopsis double mutants defective in both the PGR5/PGRL1- and NDH-dependent CEF show severe mutant phenotypes in photosynthesis and plant growth even at a very low light intensity (Munekage et al., 2004). The most straightforward explanation for this synergistic phenotype is that NDH-dependent pathway contributes to *pmf* formation redundantly with the PGR5/PGRL1-dependent pathway (Wang et al., 2015; Yamori and Shikanai, 2016). Thus, the physiological functions of PGR5/PGRL1 pathway and the NDH complex are likely different, which may depend on plant species, plant age and environmental conditions.

The functions of PSI-CEF are generally considered to be coupled tightly to the generation of pH across the thylakoid membrane under conditions where LEF is not able to generate sufficient pH (Yamori and Shikanai, 2016). Increased pH by PSI-CEF drives more ATP synthesis per NADP reduced, thereby increasing the ATP/NADPH production ratio (Yamori et al., 2015; Yamamoto and Shikanai, 2019). Since the D1 protein synthesis and repair process of damaged PSII require a large amount of ATP in a short time (Allakhverdiev et al., 2005), the CEF generation of ATP is able to promote D1 protein synthesis (Steinbeck et al., 2015). As observed in the current study, the D1 protein was obviously degraded during cold stress in both *PGR5/PGRL1A*- and *NDHM*-silenced plants compared to the control plants (pTRV; Figure 6A). When the control plants were exposed to L-R/FR conditions during cold stress, the degradation of D1 protein was significantly alleviated. However, the alleviation capability of L-R/FR on degradation of D1 protein almost disappeared in both *PGR5/PGRL1A*- and *NDHM*-silenced plants (Figure 6A). These results suggest that L-R/FR -induced cold tolerance partially depends on CEF-mediated D1 protein synthesis. Usually, generation of ΔpH via CEF can activate qE in some stress conditions (Huang et al., 2012; Zivcak et al., 2013). In our study, L-R/FR also increased qE and NPQ in the control plants (pTRV) after a cold stress, but not in the tomato *PGR5/PGRL1A*- and *NDHM*-silenced plants (Figures 6B,C). One pH sensor for qE induction is PsbS, which constitutively accumulates in the thylakoid membrane (Li et al., 2002, 2004b). The protonation of PsbS and conversion of xanthophyll carotenoids rapidly induce qE in Arabidopsis (Niyogi et al., 1998). We found that L-R/FR induced the PsbS protein accumulation and enhanced the de-epoxidation state of the xanthophyll cycle [i.e., high ratio of (A + Z)/(V + A + Z)] in tomato pTRV plants after a cold stress, but not in *PGR5/PGRL1A*- and *NDHM*-silenced plants (Figures 6D,E). Additionally, the rise in ΔpH due to CEF activation under saturating light conditions can control the electron flow from PSII to PSI via the Cyt *b_6_/f* complex (Kramer et al., 2004; Suorsa et al., 2012; Shikanai, 2014), thereby protecting PSI from photodamage (Tikkanen and Aro, 2014). Recent studies have clearly shown that PGR5-dependent CEF protects PSI under fluctuating light by downregulating electron transport through the Cyt *b_6_/f* complex (donor-side regulation) and adjusting the ATP/NADPH production ratio (acceptor-side regulation; Yamamoto and Shikanai, 2019). Furthermore, CEF activation under high light can protect PSI through preventing the overreduction of the PSI acceptor side and the production of ROS at the acceptor side of PSI (Munekage et al., 2002; Suorsa et al., 2012). In support of this, we observed that L-R/FR-induced activities of antioxidant enzymes were abolished in the tomato *PGR5/PGRL1A*- and *NDHM*-silenced plants (Figure 7). These results demonstrate that the involvement of PGR5/PGRL1A and NDHM in the induction of ROS scavenging, qE, NPQ, (A + Z)/(V + A + Z) ratio, PsbS and D1 proteins accumulation, which emphasizes the dual roles of CEF in FR induction of photoprotection during cold stress.

## Conclusion

**FIGURE 8:**
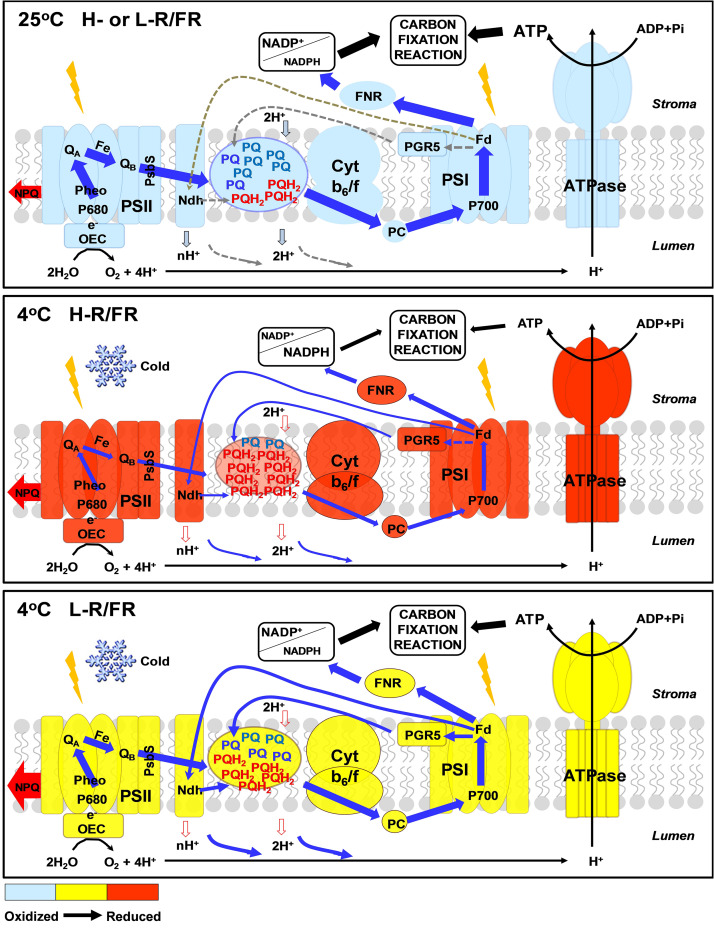
Proposed mechanisms for far-red light alleviating overreduction of the entire intersystem electron transfer chain during cold stress. Low temperature destroys the balance between light reactions and electron consumption in the chloroplast, leading to an overreduction of electron carriers, such as Q_A_^–^, Q_B_^–^ reducing centers and PQ. During cold stress, L-R/FR activates the PGR5/PGRL1- and NDH-dependent CEF in tomato plants from PSI to the PQ pool without net NADPH production and generates ΔpH across the thylakoid membrane via the Q cycle in the Cyt *b_6_f* complex. In the PSI acceptor-side regulation, *pmf* composed of ΔpH and Δψ drives ATP synthesis via ATP synthase and adjusts the ATP/NADPH production ratio, eventually, alleviating the PSI acceptor-side limitation of electron transport by increasing electron sink capacity downstream of PSI. In the PSI donor-side regulation, luminal acidification slows plastoquinol oxidation at the Cyt *b_6_f* complex to prevent excess electron flow toward PSI. In addition, L-R/FR induced luminal acidification induces qE quenching in the PSII antenna to discard excess photon energy as heat. FNR, Fd:NADP^+^ oxidoreductase; PC, plastocyanin. The degree of oxidized and reduced electron carries are indicated by color from blue to red. The thickness of blue and red color arrows represents the strength of electron flows and NPQ, respectively.

On the basis of all the results described above, we proposed the following model (Figures 8A–C). Low temperature destroys the balance between light reactions and electron consumption in the chloroplast, leading to an overreduction of electron carriers, such as Q_A_^–^, Q_B_^–^ reducing centers and PQ (Figure 8B). L-R/FR activates the PGR5/PGRL1A- and NDH-dependent CEF in tomato plants during cold stress, which alleviates cold-induced photoinhibition via three pathways (Figure 8C). (1) L-R/FR –induced CEF downregulates the Cyt *b_6_f* complex activity for slowing down the electron flow toward PSI, alleviates PSI acceptor-side limitations and overreduction of electron carriers by promoting NADP^+^/NADPH ratio and ROS scavenging; (2) L-R/FR –induced CEF promotes the protonation of PsbS and conversion of xanthophyll carotenoids to rapidly activate qE and NPQ for thermal dissipation; (3) L-R/FR –induced CEF generates ATP to promote D1 protein synthesis for repairing the damaged PSII. The roles of FR light have been largely neglected for the “red drop.” Our results clearly show that FR light should be taken into account in future photosynthetic studies (Kono et al., 2017; Zhen et al., 2018), especially ecophysiological studies seeking a mechanistic understanding of the relationship between light and temperature in plant performance in nature.

## Data Availability Statement

All datasets generated for this study are included in the article/Supplementary Material.

## Author Contributions

FW and TL designed the research. FW, JY, XW, XB, HX, YBL, and JL performed the experiments. FW, YFL, HQ, and MQ analyzed the data. FW, GA, and TL wrote and revised the manuscript.

## Conflict of Interest

The authors declare that the research was conducted in the absence of any commercial or financial relationships that could be construed as a potential conflict of interest.

## Footnotes

1 http://tgrc.ucdavis.edu
