# Supplementary material for: PGR5/PGRL1 and NDH Mediate Far-Red Light-Induced Photoprotection in Response to Chilling Stress in Tomato
Source: Front Plant Sci. 2020 May 27;11:669. doi: 10.3389/fpls.2020.00669 (PMC7270563; doi:10.3389/fpls.2020.00669)
Supplement: Supplementary file 1 [file Data_Sheet_1.PDF]

TABLE S1 | Primer sequences used for vector construction and qRT-PCR analysis.

Gene	Accession number	Forward primer (5'-3')	Reverse primer (5'-3')	
PGR5	Solyc09g090570	ATCAACTTAGGGGCAAAGCT	CCTTTGCTTCTGATCTGCTCC	qRT-PCR
PGRL1A	Solyc08g080050	CGATGATTTGACTGGATTCG	CCACAATATGAAGGGCAATG	qRT-PCR
PGRL1B	Solyc08g007770	CGATTTGACTGGATTCGAGA	AACAATGGCGTTTGTGATTG	qRT-PCR
NDHM	Solyc04g057980	CTCCATTGACGGAGTACACG	ACCCAACTCTTGGTTTCCC	qRT-PCR
NDHB	Solyc09g083190	CATTTCATGGCTGTTTCCTCC	TGTGCTTCACTTGGTTTGTCC	qRT-PCR
NDHH	Solyc07g032430	ATGTGAGAGTTGAAGCCCCA	ACATGGTGGGCGAATTTTCC	qRT-PCR
ACTIN	Solyc11g005330	TGTCCCTATTTACGAGGGTTATGC	CAGTTAAATCACGACCAGCAAGAT	qRT-PCR
PGR5	Solyc09g090570	CGgaattcGCCTCTTCATTACCACTCA	CCGctcgagCCCATCATTATCCTTGATCTCACTG	VIGS
PGRL1A	Solyc08g080050	CCGgaattcTCAAGAACAAGAATGGCCACC	CGCggatccCACTGCTCCCTTCCCACATA	VIGS
NDHM	Solyc04g057980	CGgaattcGATCCCGAAACCTTTGCTT	CCGctcgagTCCATTGTAATTGAACCCA	VIGS

TABLE S2 | Definition of terms and formulae for calculation of the JIP-test parameters from the Chl a fluorescence transient OJIP emitted by dark-adapted leaves.

Fluorescence parameters	Description
$\psi_0 \equiv T_0/TR_0 = (1 - V_J)$	probability (at t = 0) that a trapped exciton moves an electron into the electron transport chain beyond Q_A^-
$\phi_{Eo} \equiv ET_0/ABS = [1 - (F_0/F_M)]\ \psi_0$	quantum yield of electron transport (at t = 0)
$\phi_{Po} \equiv TR_0/ABS = [1 - (F_0/F_M)]$	maximum quantum yield of primary photochemistry (at t = 0)
$V_I = (F_I - F_0)/(F_M - F_0)$	relative variable fluorescence at time I-step ($F_I \equiv F_{30ms}$, fluorescence at the 30 ms of O-J-I-P)
$1\text{-}V_I = 1\text{-}(F_I - F_0)/(F_M - F_0)$	reflects the content of PSI reaction centers as well as the electron flow capacity from plastoquinone to the PSI electron acceptors
$V_K = (F_{300} - F_0)/(F_M - F_0)$	relative variable fluorescence at time K-step ($F_{300\mu s}$, fluorescence at 300 µs)
$V_J = (F_J - F_0)/(F_M - F_0)$	relative variable fluorescence at time J-step ($F_J \equiv F_{2ms}$, fluorescence at the 2 ms of O-J-I-P)
$V_K/V_J = [(F_{300} - F_0)/(F_M - F_0)]/[(F_J - F_0)/(F_M - F_0)]$	a relative measure of inactivation of OEC and/or the functional antenna size
$OEC = [1\text{-}(V_K/V_J)_{treat}]/[1\text{-}(V_K/V_J)_{control}]\times 100$	the fraction of O_2 evolving centre (OEC)
$M_0 \equiv (\Delta V/\Delta t)_0 \equiv 4\ (F_{300\mu s} - F_0)/(F_M - F_0)$	approximated initial slope (in ms^{-1}) of the fluorescence transient V = f(t)
$\phi_{Ro} = (1 - F_0/F_M)(1 - V_I)$	quantum yield for reduction of end electron acceptors at the PSI acceptor side (RE)
$\delta_{Ro} = (1 - V_I)/(1 - V_J)$	efficiency/probability with which an electron from the intersystem electron carriers moves to reduce end electron acceptors at the PSI acceptor side (RE)
$ABS/RC = M_0\ (1/V_J)\ (1/\phi_{Po})$	absorption flux per RC
$ABS/CS_M \approx F_M$	absorption flux per CS, approximated by F_M
$TR_0/CS_M = \phi_{Po}\ (ABS/CS_M)$	trapped energy flux per CS (at t=0)
$ET_0/CS_M = \phi_{Eo}\ (ABS/CS_M)$	electron transport flux per CS (at t = 0)
$DI_0/CS_M = (ABS/CS_M) - (TR_0/CS_M)$	dissipated energy flux per CS (at t = 0)
$RC/CS_M = \phi_{Po}\ (V_J/\ M_0)\ (ABS/CS_M)$	density of RCs (Q_A^- reducing PSII reaction centers)
$PI_{ABS} \equiv (RC/ABS)\ [\phi_{Po}/(1 - \phi_{Po})]\ [\psi_0/(1 - \psi_0)]$	performance index (potential) for energy conservation from photons absorbed by PSII to the reduction of intersystem electron acceptors
$PI_{total} = (PI_{ABS}) \times [\delta_{Ro}/(1 - \delta_{Ro})]$	performance index (potential) for energy conservation from photons absorbed by PSII to the reduction of PSI end acceptors
