# Supplementary material for: PGR5/PGRL1 and NDH Mediate Far-Red Light-Induced Photoprotection in Response to Chilling Stress in Tomato
Source: Front Plant Sci. 2020 May 27;11:669. doi: 10.3389/fpls.2020.00669 (PMC7270563; doi:10.3389/fpls.2020.00669)
Supplement: Supplementary file 2 [file Presentation_1.pdf]

Supplementary Figures

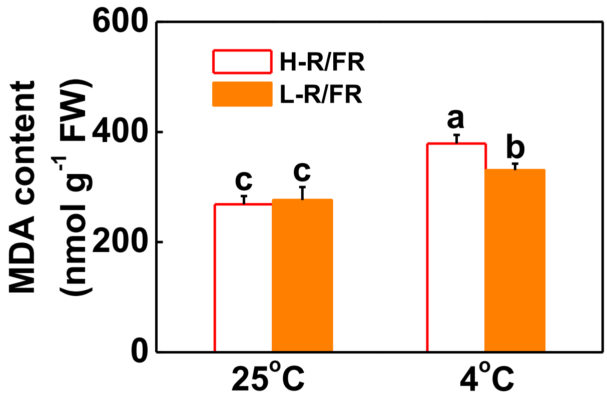

FIGURE S1 | The MDA content in tomato plants after cold stress. MDA was measured in plants grown at high red to far-red light ratios (H-R/FR, 2.5) or low red to far-red light ratios (L-R/FR, 0.5) conditions after exposed to 25 °C or 4 °C for 5 d. For light-quality treatments, plants were maintained at R conditions (120 μmol m^{-2} s^{-1}) and supplemented with different intensities of FR. Data are presented as the means of three biological replicates (±SD).

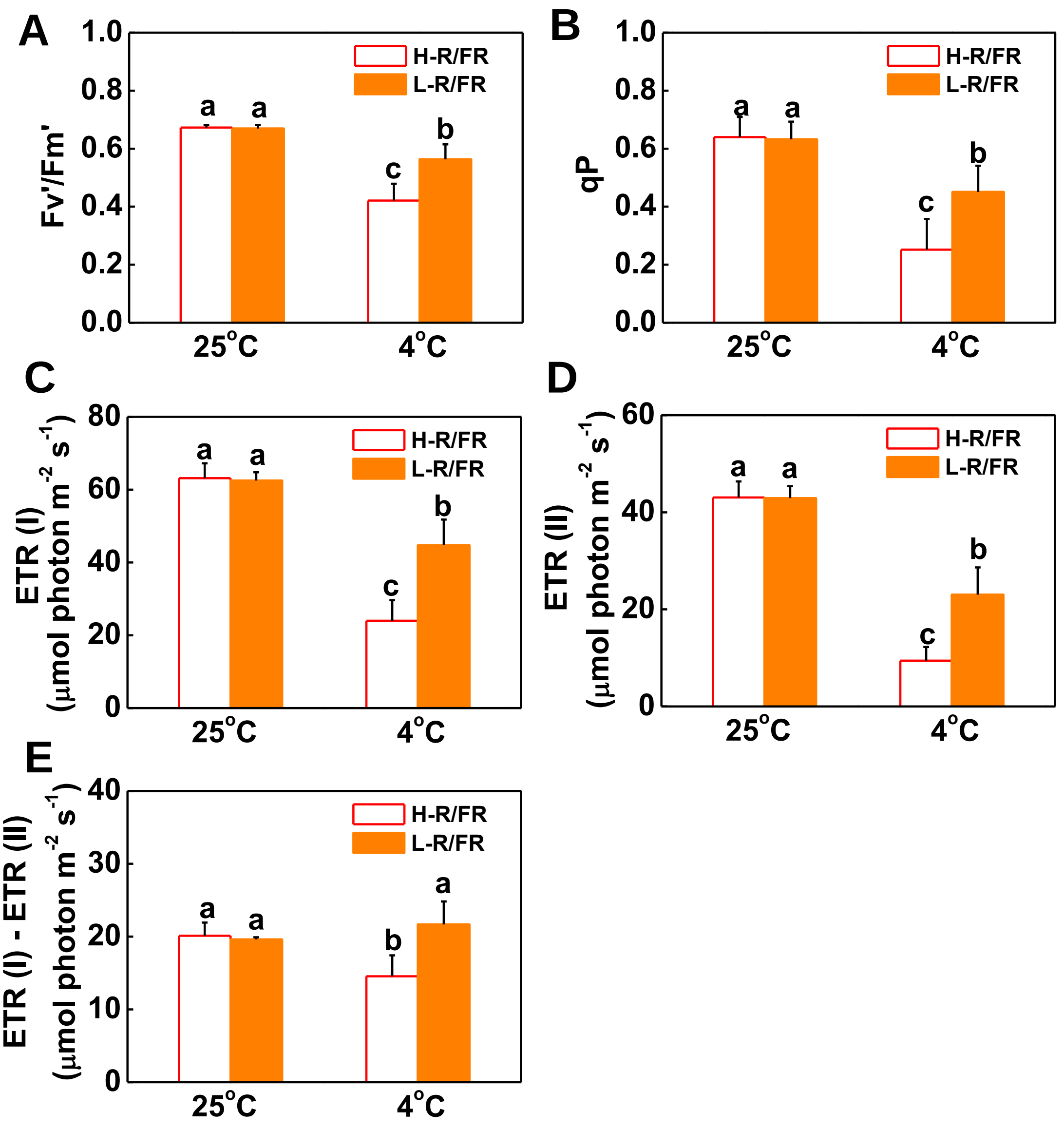

FIGURE S2 | Roles of light-quality in the electron absorbed, transport and energy distribution in photosynthetic response under cold stress. **(A-E)** Fv'/Fm' **(A)**, qP **(B)**, ETR (II) **(C)**, ETR(I) **(D)** and ETR(I)-ETR(II) **(E)** in tomato plants grown at high red to far-red light ratios (H-R/FR, 2.5) or low red to far-red light ratios (L-R/FR, 0.5) conditions after exposed to 25 °C or 4 °C for 5 d. For light-quality treatments, plants were maintained at R conditions (120 μmol m^{-2} s^{-1}) and supplemented with different intensities of FR. Data are presented as the means of three biological replicates (±SD). Different letters indicate significant differences ($P < 0.05$) according to Tukey's test.

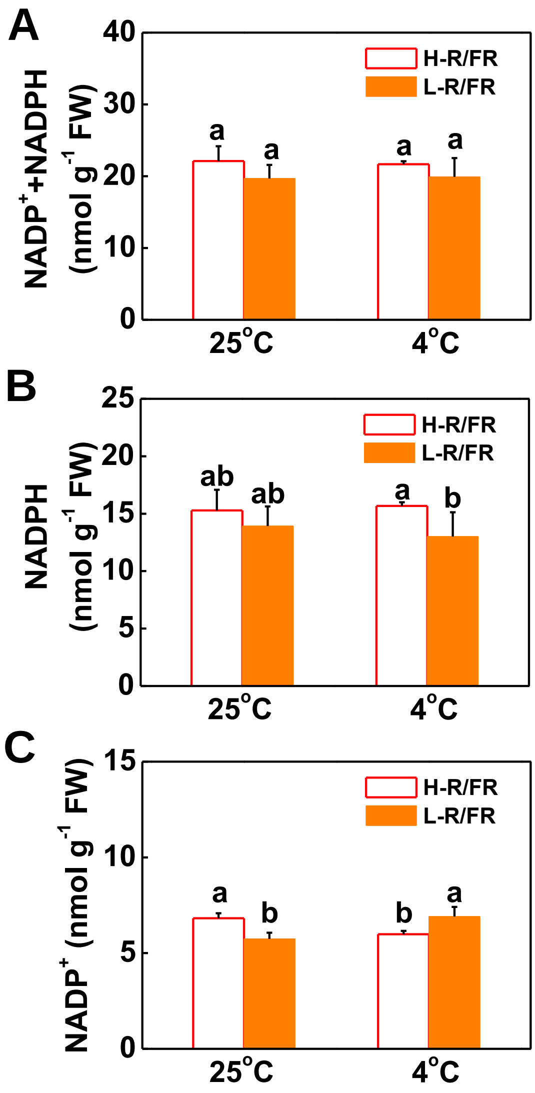

FIGURE S3 | Effects of light-quality on the production of $NADP^+$ and NADPH in tomato plants under cold stress. **(A-C)** The production of $NADP^+$ + NADPH **(A)**, NADPH **(B)** and $NADP^+$ **(C)** in tomato plants grown at high red to far-red light ratios (H-R/FR, 2.5) or low red to far-red light ratios (L-R/FR, 0.5) conditions after exposed to 25 °C or 4 °C for 5 d. For light-quality treatments, plants were maintained at R conditions (120 μmol m^{-2} s^{-1}) and supplemented with different intensities of FR. Data are presented as the means of three biological replicates (±SD). Different letters indicate significant differences ($P < 0.05$) according to Tukey's test.

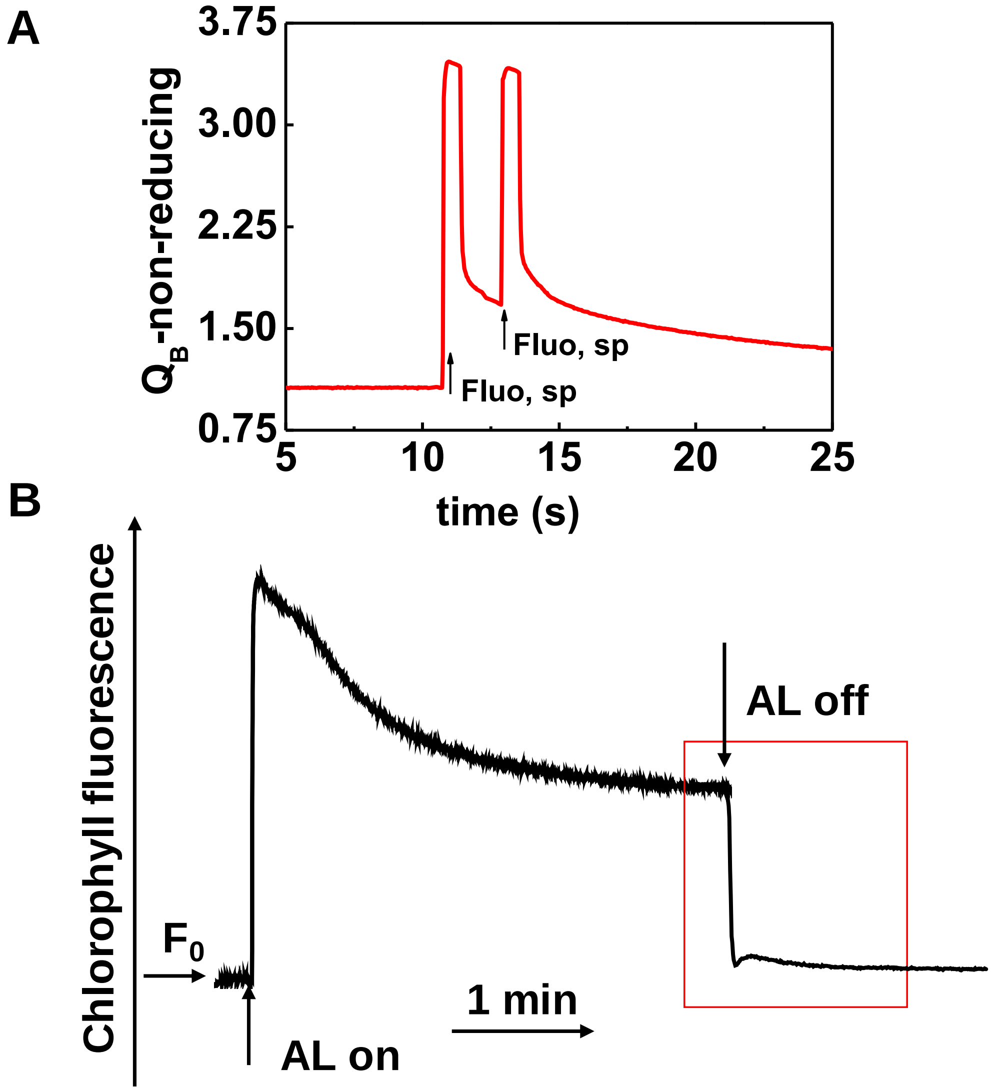

FIGURE S4 | Monitoring method of Q_B-non-reducing side of PSII and post-illumination chlorophyll fluorescence transients. **(A)** In the double hit method two fluorescence transients were induced by two subsequent pulses (each of 1 s duration). The first pulse (denoted as 1st hit) was conducted after a dark period long enough to ensure the reopening of all reaction centers, followed by a second pulse (2nd hit). The duration of the dark interval between two hits is 500 ms. **(B)** Typical induction kinetics of chlorophyll fluorescence in a dark-adapted leaf of tomato wild type under illumination with actinic light (AL; 250 mmol photons m^{-2} s^{-1}) are shown. After switching the light off (AL off), transient increases in chlorophyll fluorescence were recorded under low nonactinic light.

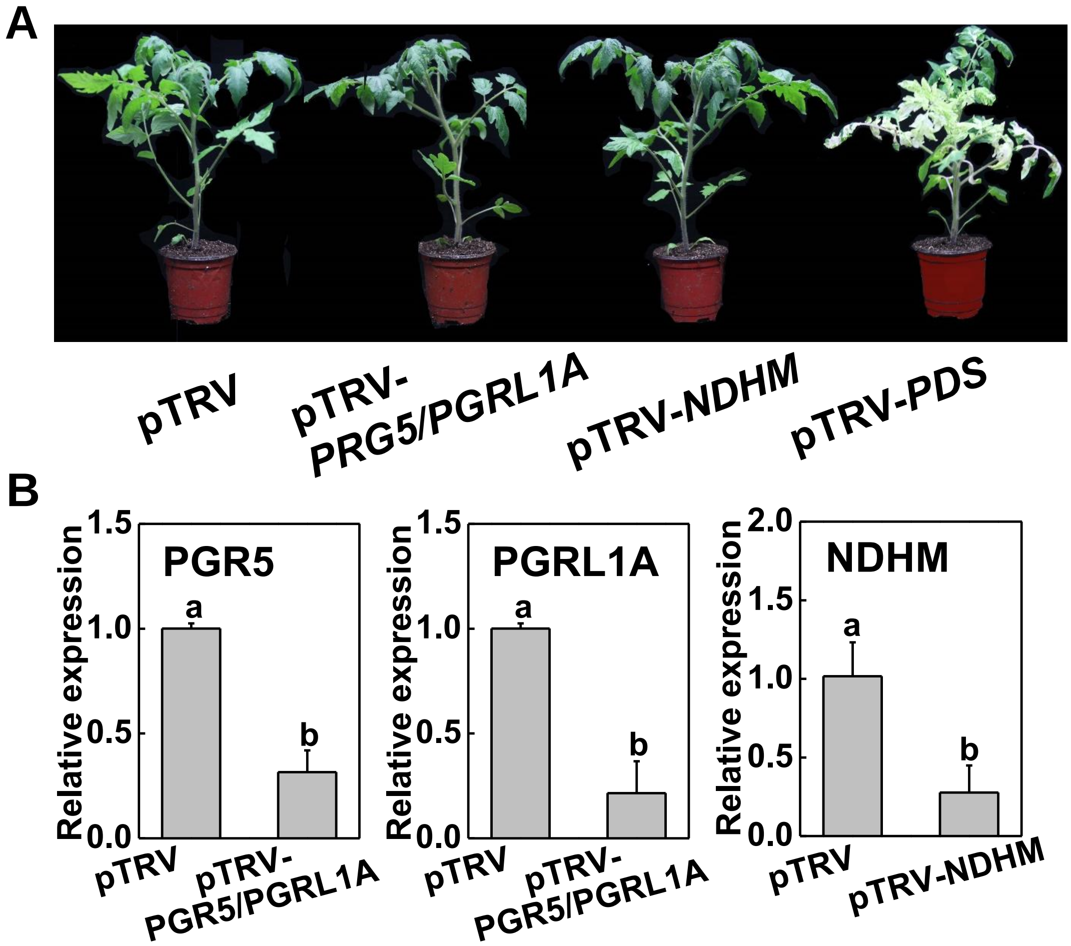

FIGURE S5 | Phenotypes and silencing efficiency of *PGR5* and *PGRL1A* co-silenced plants, and *NdhM*-silenced plants. **(A)** Phenotypes of wild-type (pTRV), *PGR5* and *PGRL1A* co-silenced plants (pTRV-*PGR5/PGRL1A*), and *NDHM*-silenced plants (pTRV-*NDHM*). *PDS* (phytoene desaturase gene, a gene involved in chlorophyll biosynthesis)-silenced (pTRV-*PDS*) tomato plants was used to monitor the virus-induced gene silencing (VIGS) progression. Photographs were taken 4 weeks after TRV infection. **(B)** Relative expression of *PGR5* and *PGRL1A* in pTRV-*PGR5/PGRL1A* tomato plants, and *NDHM* in pTRV-*NDHM* tomato plants were calculated using the pTRV plants as 100%. Samples are from the 4th leaf of six silenced plants. Data are presented as the means of three biological replicates (±SD). Different letters indicate significant differences ($P < 0.05$) according to Tukey's test.

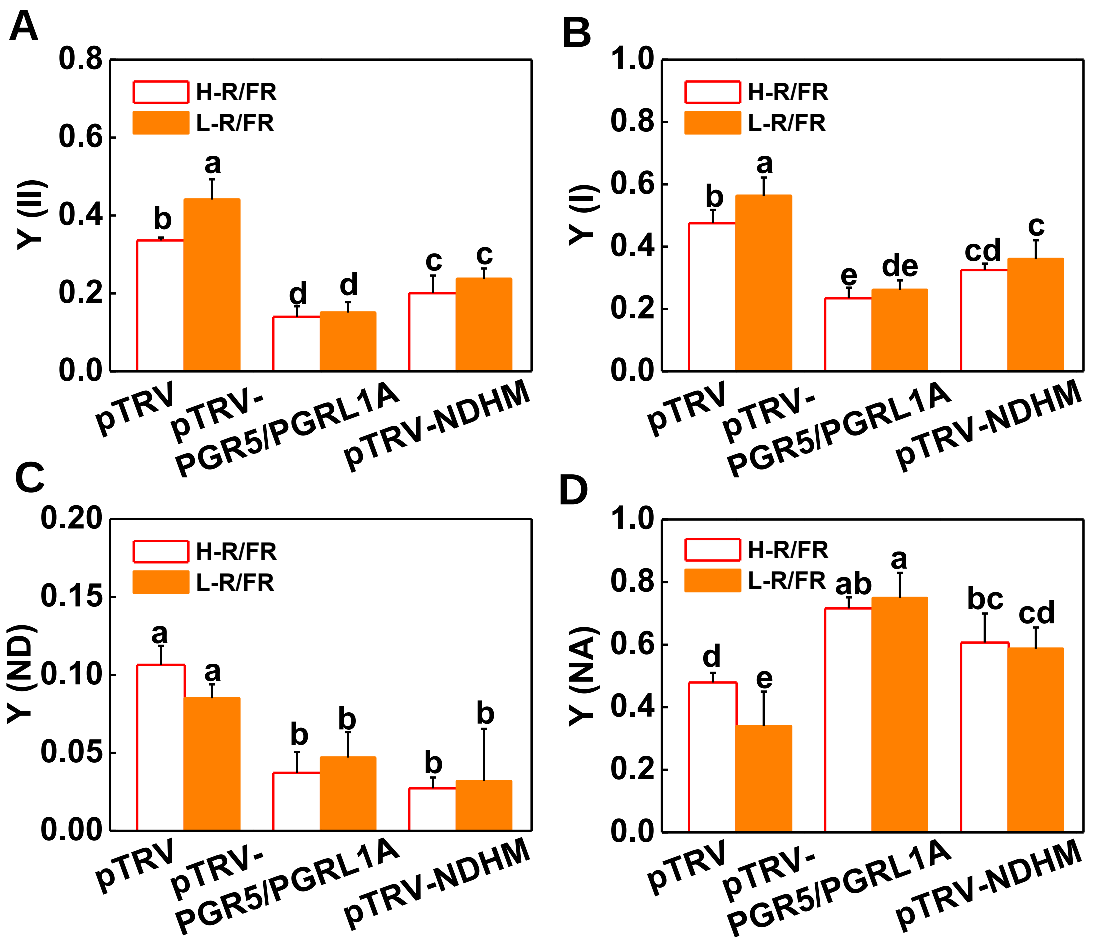

FIGURE S6 | The light-adapted PSI and PSII parameters in WT, *PGR5/PGRL1A*- and *NDHM*-silenced plants under different light quality after cold stress. **(A-D)** Y(II) **(A)**, Y(I) **(B)**, Y(ND) **(C)** and Y(NA) **(D)** in wild-type (pTRV), *PGR5* and *PGRL1A* co-silenced plants (pTRV-*PGR5/PGRL1A*), and *NDHM*-silenced plants (pTRV-*NDHM*).tomato plants grown at high red to far-red light ratios (H-R/FR, 2.5) or low red to far-red light ratios (L-R/FR, 0.5) conditions after exposed to 4 °C for 7 d. For light-quality treatments, plants were maintained at R conditions (120 μmol m^{-2} s^{-1}) and supplemented with different intensities of FR. Data are presented as the means of three biological replicates (±SD). Different letters indicate significant differences (P < 0.05) according to Tukey's test.
